# Crystal structure and Hirshfeld surface analysis of a pyrrolo-thia­zine complex

**DOI:** 10.1107/S2056989021006642

**Published:** 2021-07-02

**Authors:** R. Sribala, N. Srinivasan, P. Rajalaksmi, S. Indumathi, R.V. Krishnakumar

**Affiliations:** a Department of Physics, Thiagarajar College, Madurai - 625 009, India; b Department of Physics, M.G.R College, Hosur - 635130, India; c School of Chemistry, Madurai Kamaraj University, Madurai - 625 021, India

**Keywords:** crystal structure, pyrrolo derivatives, thia­zine, hydrogen bonding, Hirshfeld surface analysis, two-dimensional fingerprint plots

## Abstract

In the title compound, diethyl 2,2-dioxo-4-(thio­phen-2-yl)-1-[(thio­phen-2-yl)meth­yl]-3,4,6,7,8,8a-hexa­hydro-1*H*-pyrrolo­[2,1-*c*][1,4]thia­zine-1,3-di­carboxyl­ate, C_22_H_28_NO_6_S_3_, the pyrrolo ring is in an envelope conformation while the thia­zine ring adopts a near chair conformation. The dihedral angles between the thia­zine ring and the methyl­thienyl, thienyl and pyrrolo rings are 64.0 (2), 87.92 (7) and 5.6 (2)°, respectively. In the crystal, the mol­ecules are linked by weak C—H⋯O hydrogen bonds. A Hirshfeld surface analysis was performed to investigate the inter­molecular inter­actions. Disorder of the methyl­thienyl group with site occupancies of 0. 792 (3) and 0.208 (3) is observed

## Abstract 2

In diethyl 2,2-dioxo-4-(thio­phen-2-yl)-1-[(thio­phen-2-yl)meth­yl]-3,4,6,7,8,8a-hexa­hydro-1*H*-pyrrolo­[2,1-*c*][1,4]thia­zine-1,3-di­carboxyl­ate, the pyrrolo ring is in an envelope conformation while the thia­zine ring adopts a near chair conformation. The dihedral angles between the thia­zine ring and the methyl­thienyl, thienyl and pyrrolo rings are 64.0 (2), 87.92 (7) and 5.6 (2)°, respectively. In the crystal, the mol­ecules are linked by weak C—H⋯O hydrogen bonds.

## Chemical context   

Heterocyclic compounds play a vital role in modern drug discovery and are used to generate novel frameworks with potential bioactivity. They are prevalent in nature and play a vital role in the metabolism of all living things, being utilized at almost every stage of the many biochemical processes necessary to sustain life. Heterocycles actively participate in various inter­molecular inter­actions, metal coordination bonds, hydro­phobic forces *etc*. The assimilation of heteroatoms within a carbon ring system can be used to explore different avenues for biologically active heterocycles, which have increasing importance in pharmacological activities. The crystal structures of sulfur-containing heterocycles and their supra­molecular features are of significant inter­est in the development of anti-cancer drugs. The role of the sulfur atom in biological system, *viz*. regulation translation *via* the sulfuration of transfer RNA is noteworthy. Majority of the anti-cancer drugs in the pharma industry are built with heterocycles as primary structural components. 
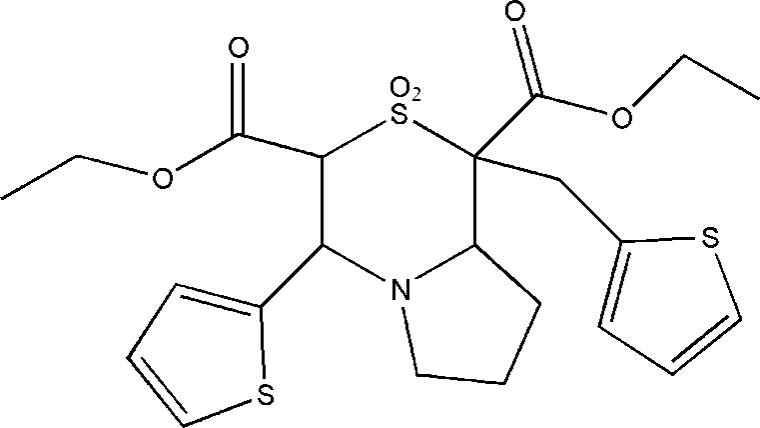


Thia­zine derivatives are the most important source of biologically active heterocyclic compounds. They exhibit anti­microbial and anti-diabetic (Faidallah *et al.*, 2011 ▸; Adly, 2012 ▸), anti-histaminic (Arya *et al.*, 2012 ▸), anti­bacterial and anti­fungal (Tandon *et al.*, 2006 ▸; Zia-ur-Rehman *et al.*, 2009 ▸; Ganorkar *et al.*, 2013 ▸), antagonistic (Galanski *et al.*, 2006 ▸), anti­oxidant (Smith, 1951 ▸), analgesic and anti-inflammatory, (Chia *et al.*, 2008 ▸; Tozkoparan *et al.*, 2002 ▸) anti-tuberculosis (Koketsu *et al.*, 2002 ▸), anti­tumor (Wang *et al.*, 2012 ▸)), anti­mycobacterial (Indumathi *et al.*, 2009 ▸) and anti­helminthic and insecticidal (Smith, 1942 ▸) activity and act as potassium channel-opening agents (Erker *et al.*, 2000 ▸), nitric oxide synthase inhibitors (Tung-Mei *et al.*, 2005 ▸), smooth muscle relaxants (Schreder *et al.*, 2000 ▸), urokinase inhibitors (Tanaka *et al.*, 1998 ▸) and as as myocardial calcium channel modulators (Budriesi *et al.*, 2002 ▸). Besides, thia­zine derivatives are effective corrosion inhibitors for carbon steel in acidic media. They thus represent an inter­esting class of heterocyclic compound worthy of further exploration.

Pyrrolo derivatives have pharmacological activities such as anti-inflammatory, cytotoxicity against a variety of marine and human tumour models (Dannhardt *et al.*, 2000 ▸; Evans *et al.*, 2003 ▸) and are used in the treatment of hyperlipidemia (Holub *et al.*,2004 ▸). One of the tris­ubstituted pyrrole, porphobilinogen, serves as a biosynthetic precursor to many natural products including heme, the red pigment in haemoglobin (Cox *et al.*, 2008 ▸). In view of these observations and in a continuation of our work (Sribala *et al.*, 2018 ▸) on novel heterocycles of pharmaceutical importance, the crystal structure of the sulfur-containing heterocycle, diethyl 2,2-dioxo-4-(thio­phen-2-yl)-1-[(thio­phen-2-yl)meth­yl]-3,4,6,7,8,8a-hexa­hydro-1*H*-pyrrolo­[2,1-*c*][1,4]thia­zine-1,3-di­carboxyl­ate is des­cribed herein. Hydrogen-bonding inter­actions in the title compound were substanti­ated with the aid of Hirshfeld surface analysis.

## Structural commentary   

**Figure 1:**
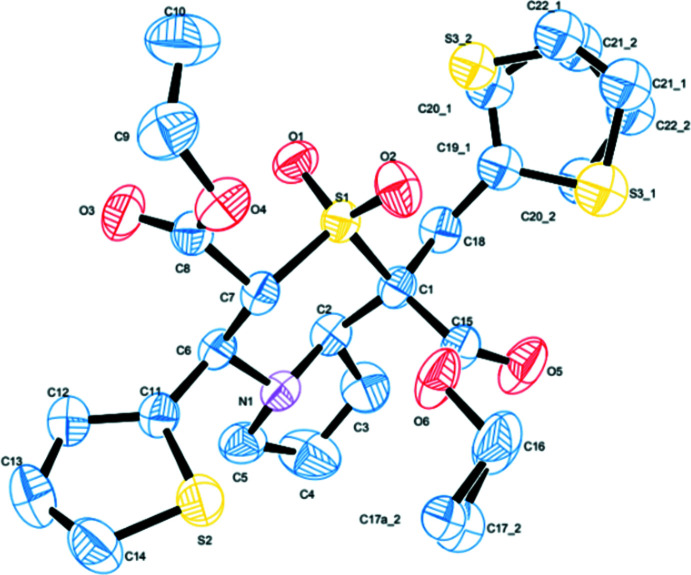
The mol­ecular structure of the title compound, with atom labels and 50% probability displacement ellipsoids for non-H atoms. H atoms have been omitted for clarity.

The title compound (Fig. 1 ▸) crystallizes in the triclinic system with a centrosymmetric space group *P* 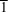 . The thienylmethyl group shows an unexpected geometry, suggesting a ring-flip disorder where two sets of atomic sites with disorder components are related by an approximate 180° rotation about the exocyclic C—C bond. A conformational analysis of the five-membered pyrrolo ring (N1/C2/C3/C4/C5) [puckering parameters *Q*(2) = 0.386 (3) Å and φ(2) = 0.6 (6)°] indicates an envelope conformation on the nitro­gen atom (N1). The six-membered thia­zine ring adopts a near chair conformation with puckering parameters *Q* = 0.607 (2) Å, θ = 171.65 (19)° and φ = 306.1 (14)°. The dihedral angle between the planes of the thia­zine (S1/C1/C2/N1/C6/C7; r.m.s. deviation = 0.2475 Å) and methyl­thienyl rings (S3/C19/C20/C21/C22) is 64.0 (2)°. The thia­zine ring subtends a dihedral angle of 87.92 (7)° with the thienyl ring (S2/C11/C12/C13/C14). The dihedral angle between the planes of the thia­zine and pyrrolo rings is 5.6 (2)°, which is slightly lower than that reported in diethyl 1-(4-chloro­benz­yl)-4-(4-chloro­phen­yl)-2,2-dioxo-3,4,6,7,8,8a-hexa­hydro-1*H*-pyrrolo­[2,1-*c*][1,4]thia­zine-1,3-di­carbox­ylate and diethyl 1-(4-meth­ylbenz­yl)-4-(4-meth­ylphen­yl)-2,2-dioxo-3,4,6,7,8,8a-hexa­hydro-1*H*-pyrrolo­[2,1-*c*][1,4]thia­zine-1,3-di­carbox­ylate, hereafter referred to as compounds (I) and (II) [6.68 (10) and 8.06 (11)°; Sribala *et al.*, 2018 ▸). The terminal methyl carbon atom C10 deviates from the plane involving carboxyl group (C7/C8/O3/O4/C9) by 1.431 (3) Å [1.371 (3) and 1.409 (3) Å in compounds (I) and (II), respectively]. Similarly the major component of the disordered methyl carbon atom C17 deviates from the plane (C1/C15/O5/O6/C16) by 1.123 (12) Å. The dihedral angle between the two carboxyl planes is 55.74 (7)°, significantly different from the values obtained in (I) [12.73 (10)°] and (II) [12.07 (10)°]. The difference in value is probably due to the disorder of this atom.

## Supra­molecular features   

**Figure 2:**
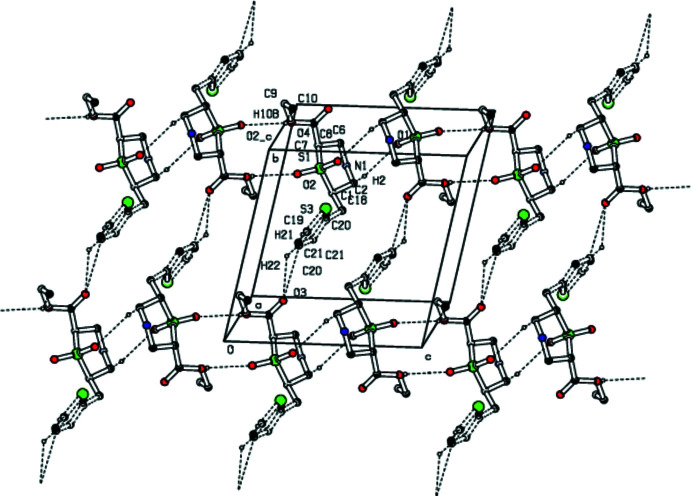
A view of the mol­ecular aggregation down the *a* axis. Ring systems and H atoms that are not involved in hydrogen bonding have been omitted for clarity.

**Figure 3:**
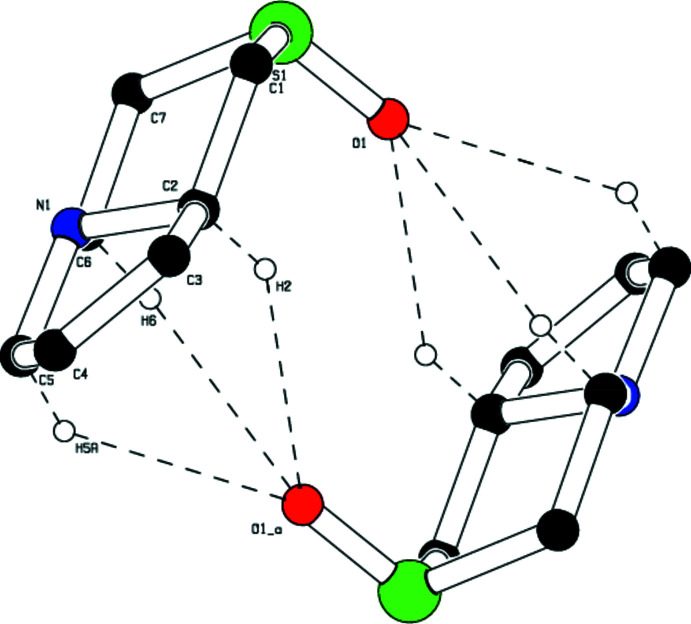
Trifurcated hydrogen bond involving donor atoms C2,C5 and C6 with oxygen atom O1 as acceptor.

**Figure 4:**
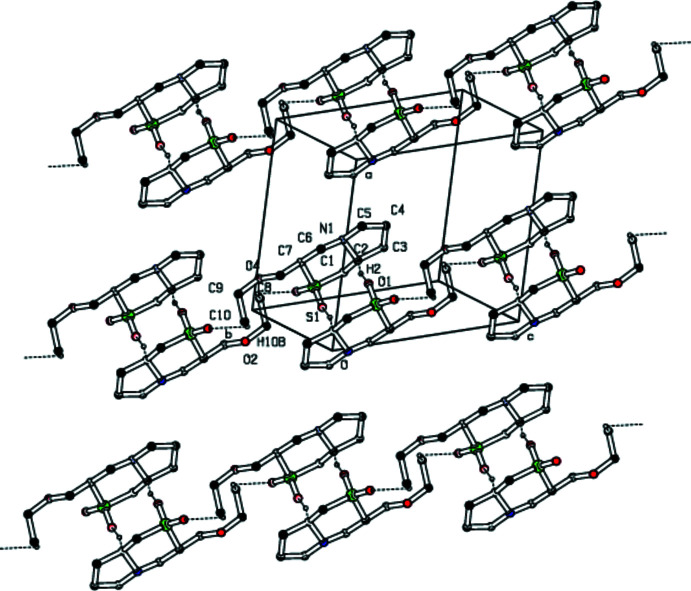
Packing of the title compound viewed along the *c* axis showing the C2—H2⋯O1 and C10—H10*B*⋯O2 hydrogen bonds forming 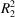 (10) and 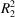 (16)rings. Dotted lines indicate hydrogen bonds. Non-participating H atoms, methyl C atoms and S atoms have been omitted for clarity.

In the crystal, mol­ecules are linked by weak C—H⋯O hydrogen bonds, forming a two-dimensional network (Table 1 ▸, Fig. 2 ▸). A rare trifurcated hydrogen-bond formation is observed involving donor atoms C2, C5 and C6 with oxygen O1 as acceptor (Fig. 3 ▸). The C10—H10*B*⋯O2^i^ inter­action leads to the formation of an 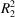 (16) graph-set motif while C2—H2⋯O1 generates an 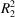 (10) motif (Fig. 4 ▸). Thus the thia­zine ring plays a dominant role in the structural cohesion *via* weak C—H⋯O hydrogen bonds. A parallel displaced π–π stacking inter­action is observed with *Cg*2⋯*Cg*2(−*x*, 1 − *y*, 1 − *z*) = 4.668 (3) Å and a slippage of 2.794 Å, where *Cg*2 is the centroid of the thienyl ring (C19/C20/C21/C22/S3).

## Hirshfeld surface analysis   

Hirshfeld surface analysis is a tool to encapsulate and visualize the inter­molecular inter­actions of a crystal on a three-dimensional surface. The mol­ecular inter­actions on the isosurface are determined using the parameters *d*_i_ and *d*_e_ (representing the distances from a given point on the surface to the nearest atom inside and outside the surface, respectively), which in turn add-on to provide the normalized contact distance, *d*_norm_. The Hirshfeld surfaces (Spackman *et al.*, 2009 ▸) together with decomposed fingerprint plots (McKinnon *et al.*, 2007 ▸; Tan *et al.*, 2019 ▸) for the title compound were generated using *Crystal Explorer 17.5* (Turner *et al.*, 2017 ▸).

**Figure 5:**
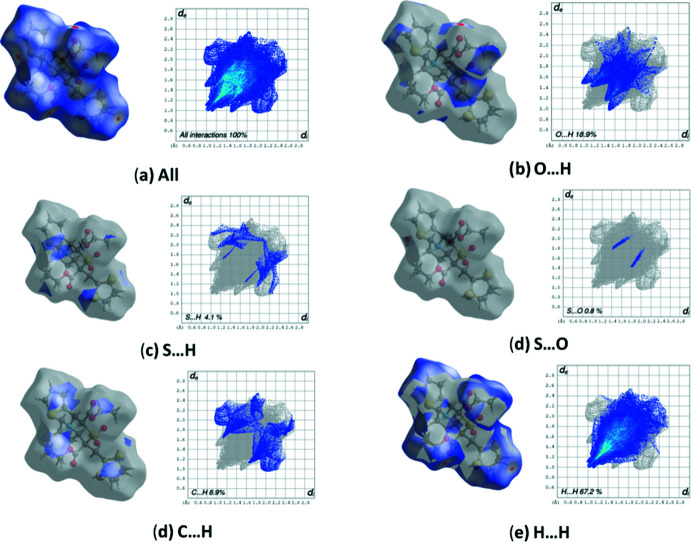
Hirshfeld surface mapped over *d*_norm_ and decomposed fingerprint plots for the dominant inter­actions.

The Hirshfeld surfaces mapped over *d*_norm_ together with decomposed fingerprint plots are presented in Fig. 5 ▸. The deep-red circular depressions represent inter­molecular short O⋯H contacts. The pale-red spots near the thienyl rings confirm the presence of C—H⋯O inter­actions, which stabilize the structure. The combined O⋯H/H⋯O inter­actions appear as large symmetrically sharp spikes at the bottom of the plot and occupy 18.9% of the total available surface. Nearly 67.2% of the total surface is captured by H⋯H short contacts resulting from the inter­action of methyl and methyl­ene hydrogens and appear as scattered points in the plot. Symmetrical wing-like projections appearing on the inter­ior side of the top of the plot result from C⋯H/H⋯C, inter­actions which represent 8.9% of the surface area. The S⋯H and H⋯S inter­actions (4.1%) appear as external sharp wings in the fingerprint plot. The least contribution comes from S⋯O contacts, accounting for only 0.8% of the Hirshfeld surface.

## Database survey   

A search in the Cambridge Structural Database (CSD Version 5.39, update of November 2017; Groom *et al.*, 2016 ▸) for the presence of pyrrolo ring organic structures having 3D coord­inates with no disorder, no ions and no other errors, with *R* factors less than 0.05 yielded 175 structures. When the search was further restricted to fused pyrrolo-thia­zine ring structures, the number of hits reduced to five, *viz*. EXIYAM (Chitradevi *et al.*, 2011 ▸), IDOGIT (Chitradevi *et al.*, 2013 ▸), NEVCUN (Indumathi *et al.*, 2007 ▸), VOKHAG (Gao, *et al.*, 2005 ▸) and SINSAM (Sribala, *et al.*, 2018 ▸) while a search for pyrrolo-thia­zine ring combined with the other substituents in skeleton of the title compound, gave zero hits.

## Synthesis and crystallization   

A mixture of ethyl 2-[(2-eth­oxy-2-oxoeth­yl)sulfon­yl]acetate (1.6 mmol), thio­phene-2-carboxaldehyde (3.2 mmol) and pyrrolidine (1.6 mmol) was dissolved in ethanol (10 mL), heated until the solution turned yellow and stirred at room temperature for 2–5 days. After completion of the reaction, the crude product was purified using flash column chromatography on silica gel (230–400 mesh) with petroleum ether and ethyl acetate mixture (95:5 *v*/*v*) as eluent (Indumathi *et al.*, 2007 ▸).

## Refinement   

Crystal data, data collection and structure refinement details are summarized in Table 2 ▸. All the hydrogen atoms were fixed using geometric HFIX constraints. H atoms were positioned geometrically (N—H = 0.98 Å, C—H = 0.93–0.98 Å) and refined using a riding model with *U*_iso_(H) = 1.2*U*_eq_(C,N) or 1.5*U*_eq_(C-meth­yl).

The title compound crystallized with disorder in the terminal carbon atom attached to one of the ethyl group in di­carboxyl­ate side chain. During refinement, although the *R* value reduced to 0.0587, a few residual peaks with significant electron density (1.17 e Å^−3^) appeared, indicating disorder in the carbon atom attached to one of the ethyl groups in the di­carboxyl­ate side chain. Hence the the terminal carbon atom C17 was split over two positions with site occupancies of 0.792 (3) and 0.208 (3) The hydrogen atoms attached to C16 are also disordered and were split using suitable HFIX constraints. All atoms in the thienyl ring (S3/C19/C20/C21/C22) were subject to rigid-bond restraints using DELU and SIMU instructions. The ring carbon atom C19 shares the same atomic site in both disorder components and was refined using EXYZ and EADP constraints. The *R* value thus reduced to 0.0439 with maximum/minimum values of residual electron densities of 0.32 and 0.56 e Å^−3^.

## Supplementary Material

Crystal structure: contains datablock(s) I. DOI: 10.1107/S2056989021006642/jy2008sup1.cif

Structure factors: contains datablock(s) I. DOI: 10.1107/S2056989021006642/jy2008Isup2.hkl

Supporting information file. DOI: 10.1107/S2056989021006642/jy2008Isup3.cml

CCDC reference: 2092264

Additional supporting information:  crystallographic information; 3D view; checkCIF report

## Figures and Tables

**Table 1:** Hydrogen-bond geometry (Å, °)

*D*—H⋯*A*	*D*—H	H⋯*A*	*D*⋯*A*	*D*—H⋯*A*
C10—H10*B*⋯O2^i^	0.96	2.63	3.227 (4)	120
C2—H2⋯O1^ii^	0.98	2.67	3.519 (3)	145
C5—H5*A*⋯O1^ii^	0.97	2.69	3.491 (3)	141
C6—H6⋯O1^ii^	0.98	2.70	3.560 (3)	146

Symmetry codes: (i) -x, -y+2, -z; (ii) -x, -y+2, -z+1.

**Table 2:** Experimental details

Crystal data	
Chemical formula	C_22_H_28_NO_6_S_3_
*M*_r_	498.66
Crystal system, space group	Triclinic, *P*\overline{1}
Temperature (K)	293
*a*, *b*, *c* (Å)	10.6886 (5), 11.4878 (5), 11.8389 (6)
α, β, γ (°)	76.007 (1), 65.584 (1), 63.213 (1)
*V* (Å^3^)	1178.87 (10)
*Z*	2
Radiation type	Mo *K*α
μ (mm^−1^)	0.35
Crystal size (mm)	0.30 × 0.20 × 0.10

Data collection	
Diffractometer	Bruker SMART APEXII CCD
Absorption correction	Multi-scan (*SADABS*; Bruker, 2009 ▸)
*T*_min_, *T*_max_	0.864, 1.0
No. of measured, independent and observed [*I* > 2σ(*I*)] reflections	21855, 4375, 3774
*R*_int_	0.021
(sin θ/λ)_max_ (Å^−1^)	0.606

Refinement	
*R*[*F* ^2^ > 2σ(*F* ^2^)], *wR*(*F* ^2^), *S*	0.044, 0.134, 1.05
No. of reflections	4375
No. of parameters	337
No. of restraints	90
H-atom treatment	H-atom parameters constrained
Δρ_max_, Δρ_min_ (e Å^−3^)	0.33, −0.56

Computer programs: *APEX2* and *SAINT* (Bruker, 2009 ▸), *SHELXS2013/1* (Sheldrick, 2008 ▸), *SHELXL2018/3* (Sheldrick, 2015 ▸), *PLUTON* (Spek, 2020 ▸) and *publCIF* (Westrip, 2010 ▸).

## supplementary crystallographic information

### Crystal data

**Table:**

C_22_H_28_NO_6_S_3_	*Z* = 2
*M**_r_* = 498.66	*F*(000) = 526
Triclinic, *P*1	*D*_x_ = 1.405 Mg m^−^^3^*D*_m_ = 1.40 Mg m^−^^3^*D*_m_ measured by floatation method
*a* = 10.6886 (5) Å	Mo *K*α radiation, λ = 0.71073 Å
*b* = 11.4878 (5) Å	Cell parameters from 6274 reflections
*c* = 11.8389 (6) Å	θ = 4.8–60.3°
α = 76.007 (1)°	µ = 0.35 mm^−^^1^
β = 65.584 (1)°	*T* = 293 K
γ = 63.213 (1)°	Needle, colorless
*V* = 1178.87 (10) Å^3^	0.30 × 0.20 × 0.10 mm

### Data collection

**Table:**

Bruker SMART APEXII CCD diffractometer	3774 reflections with *I* > 2σ(*I*)
Radiation source: fine-focus sealed tube	*R*_int_ = 0.021
φ and ω scans	θ_max_ = 25.5°, θ_min_ = 2.3°
Absorption correction: multi-scan (*SADABS*; Bruker, 2009)	*h* = −12→12
*T*_min_ = 0.864, *T*_max_ = 1.0	*k* = −13→13
21855 measured reflections	*l* = −14→14
4375 independent reflections	

### Refinement

**Table:**

Refinement on *F*^2^	90 restraints
Least-squares matrix: full	Hydrogen site location: inferred from neighbouring sites
*R*[*F*^2^ > 2σ(*F*^2^)] = 0.044	H-atom parameters constrained
*wR*(*F*^2^) = 0.134	*w* = 1/[σ^2^(*F*_o_^2^) + (0.0758*P*)^2^ + 0.5387*P*] where *P* = (*F*_o_^2^ + 2*F*_c_^2^)/3
*S* = 1.05	(Δ/σ)_max_ < 0.001
4375 reflections	Δρ_max_ = 0.33 e Å^−^^3^
337 parameters	Δρ_min_ = −0.56 e Å^−^^3^

### Special details

**Table:**

Geometry. All esds (except the esd in the dihedral angle between two l.s. planes) are estimated using the full covariance matrix. The cell esds are taken into account individually in the estimation of esds in distances, angles and torsion angles; correlations between esds in cell parameters are only used when they are defined by crystal symmetry. An approximate (isotropic) treatment of cell esds is used for estimating esds involving l.s. planes.

### Fractional atomic coordinates and isotropic or equivalent isotropic displacement parameters (Å^2^)

**Table:**

	*x*	*y*	*z*	*U*_iso_*/*U*_eq_	Occ. (<1)
S1	0.10643 (6)	0.88526 (5)	0.27014 (5)	0.04061 (16)	
S2	0.55456 (8)	0.97081 (7)	0.15698 (7)	0.0688 (2)	
O1	−0.03173 (17)	0.95752 (15)	0.36171 (15)	0.0531 (4)	
O2	0.1034 (2)	0.85385 (16)	0.16209 (15)	0.0573 (4)	
O3	0.0632 (2)	1.20507 (16)	0.24368 (18)	0.0654 (5)	
O4	0.12980 (18)	1.11430 (16)	0.06971 (14)	0.0547 (4)	
O5	0.3767 (2)	0.52543 (17)	0.2873 (2)	0.0774 (6)	
O6	0.4110 (2)	0.69543 (16)	0.16392 (17)	0.0670 (5)	
N1	0.34311 (19)	0.84775 (17)	0.37670 (16)	0.0428 (4)	
H1	0.426635	0.794818	0.309267	0.051*	
C1	0.1999 (2)	0.73789 (19)	0.35204 (19)	0.0416 (5)	
C2	0.2399 (2)	0.7824 (2)	0.44176 (19)	0.0451 (5)	
H2	0.147318	0.843315	0.497164	0.054*	
C3	0.3188 (3)	0.6708 (3)	0.5215 (3)	0.0665 (7)	
H3A	0.246738	0.656734	0.600762	0.080*	
H3B	0.375080	0.590302	0.479278	0.080*	
C4	0.4206 (4)	0.7140 (4)	0.5397 (4)	0.0880 (10)	
H4A	0.523656	0.652716	0.506363	0.106*	
H4B	0.395321	0.719435	0.627467	0.106*	
C5	0.3999 (3)	0.8470 (3)	0.4713 (2)	0.0569 (6)	
H5A	0.328281	0.916183	0.527048	0.068*	
H5B	0.494141	0.857580	0.432665	0.068*	
C6	0.2742 (2)	0.97962 (19)	0.32476 (19)	0.0406 (4)	
H6	0.187469	1.031597	0.391222	0.049*	
C7	0.2225 (2)	0.97657 (19)	0.22255 (19)	0.0402 (4)	
H7	0.311127	0.937410	0.150506	0.048*	
C8	0.1292 (2)	1.1133 (2)	0.1820 (2)	0.0447 (5)	
C9	0.0305 (3)	1.2326 (3)	0.0223 (3)	0.0683 (7)	
H9A	0.061777	1.230870	−0.066911	0.082*	
H9B	0.037761	1.307949	0.038203	0.082*	
C10	−0.1250 (4)	1.2455 (4)	0.0806 (4)	0.0922 (11)	
H10A	−0.187600	1.324389	0.047480	0.138*	
H10B	−0.132705	1.171788	0.063818	0.138*	
H10C	−0.156697	1.248790	0.168828	0.138*	
C11	0.3842 (2)	1.0435 (2)	0.2691 (2)	0.0462 (5)	
C12	0.3678 (3)	1.1617 (2)	0.2960 (2)	0.0542 (6)	
H12	0.283730	1.216203	0.354333	0.065*	
C13	0.4974 (4)	1.1879 (3)	0.2220 (3)	0.0715 (8)	
H13	0.506915	1.262846	0.227014	0.086*	
C14	0.6042 (3)	1.0954 (3)	0.1447 (3)	0.0721 (8)	
H14	0.695061	1.098978	0.090502	0.087*	
C15	0.3384 (3)	0.6402 (2)	0.2639 (2)	0.0481 (5)	
C16	0.5467 (4)	0.6097 (3)	0.0760 (3)	0.0857 (10)	
H16	0.549343	0.549916	0.033476	0.103*	0.792 (3)
H16A	0.524753	0.599733	0.008051	0.103*	0.208 (3)
H16B	0.584819	0.524079	0.117016	0.103*	0.208 (3)
C18	0.0856 (3)	0.6761 (2)	0.4312 (2)	0.0494 (5)	
H18A	0.136028	0.596702	0.474240	0.059*	0.792 (3)
H18B	0.007236	0.735721	0.494289	0.059*	0.792 (3)
H18C	0.136028	0.596702	0.474240	0.059*	0.208 (3)
H18D	0.007236	0.735721	0.494289	0.059*	0.208 (3)
S3_1	0.09583 (14)	0.50348 (10)	0.29130 (11)	0.0652 (4)	0.792 (3)
C19_1	0.0117 (2)	0.64238 (18)	0.3678 (2)	0.0478 (5)	0.792 (3)
C20_1	−0.1310 (6)	0.7043 (5)	0.3731 (7)	0.0594 (11)	0.792 (3)
H20A_1	−0.148183	0.794177	0.341102	0.071*	0.792 (3)
H20B_1	−0.195416	0.705916	0.459804	0.071*	0.792 (3)
C21_1	−0.0643 (5)	0.5378 (4)	0.2636 (5)	0.0613 (9)	0.792 (3)
H21_1	−0.071925	0.483193	0.222378	0.074*	0.792 (3)
C22_1	−0.1757 (5)	0.6500 (4)	0.3070 (5)	0.0627 (9)	0.792 (3)
H22_1	−0.267612	0.686988	0.296132	0.075*	0.792 (3)
S3_2	−0.1644 (4)	0.7422 (5)	0.3683 (7)	0.0605 (12)	0.208 (3)
C19_2	0.0117 (2)	0.64238 (18)	0.3678 (2)	0.0478 (5)	0.208 (3)
C20_2	0.0676 (16)	0.5264 (9)	0.3213 (15)	0.052 (2)	0.208 (3)
H20_2	0.156587	0.459071	0.325815	0.062*	0.208 (3)
C21_2	−0.1504 (17)	0.6172 (13)	0.301 (2)	0.059 (2)	0.208 (3)
H21_2	−0.233728	0.621400	0.289946	0.070*	0.208 (3)
C22_2	−0.0206 (14)	0.5147 (15)	0.2644 (17)	0.055 (2)	0.208 (3)
H22_2	0.006362	0.450567	0.213808	0.066*	0.208 (3)
C17_2	0.6689 (10)	0.6277 (11)	0.0637 (12)	0.203 (6)	0.792 (3)
H17A_2	0.756213	0.568897	0.005954	0.305*	0.792 (3)
H17B_2	0.680508	0.610621	0.142986	0.305*	0.792 (3)
H17C_2	0.656401	0.716174	0.033258	0.305*	0.792 (3)
C17A_2	0.661 (2)	0.6669 (18)	0.0269 (11)	0.046 (3)	0.208 (3)
H17D_2	0.625474	0.749063	−0.018241	0.069*	0.208 (3)
H17E_2	0.752574	0.608189	−0.027517	0.069*	0.208 (3)
H17F_2	0.678945	0.680424	0.094874	0.069*	0.208 (3)

### Atomic displacement parameters (Å^2^)

**Table:**

	*U*^11^	*U*^22^	*U*^33^	*U*^12^	*U*^13^	*U*^23^
S1	0.0442 (3)	0.0366 (3)	0.0397 (3)	−0.0151 (2)	−0.0150 (2)	−0.0017 (2)
S2	0.0537 (4)	0.0648 (4)	0.0817 (5)	−0.0287 (3)	−0.0006 (3)	−0.0267 (4)
O1	0.0404 (8)	0.0470 (9)	0.0600 (10)	−0.0110 (7)	−0.0119 (7)	−0.0071 (7)
O2	0.0814 (12)	0.0579 (10)	0.0484 (9)	−0.0373 (9)	−0.0312 (8)	0.0044 (7)
O3	0.0795 (12)	0.0375 (9)	0.0799 (12)	−0.0058 (8)	−0.0436 (10)	−0.0121 (8)
O4	0.0567 (10)	0.0523 (9)	0.0416 (8)	−0.0156 (8)	−0.0162 (7)	0.0049 (7)
O5	0.0702 (12)	0.0372 (9)	0.0896 (14)	−0.0071 (8)	−0.0115 (10)	−0.0044 (9)
O6	0.0648 (11)	0.0441 (9)	0.0575 (10)	−0.0150 (8)	0.0104 (8)	−0.0171 (8)
N1	0.0400 (9)	0.0393 (9)	0.0426 (9)	−0.0097 (7)	−0.0133 (7)	−0.0065 (7)
C1	0.0447 (11)	0.0363 (10)	0.0380 (10)	−0.0139 (9)	−0.0125 (9)	−0.0009 (8)
C2	0.0479 (12)	0.0436 (11)	0.0382 (10)	−0.0153 (9)	−0.0141 (9)	−0.0009 (8)
C3	0.0843 (19)	0.0615 (16)	0.0626 (16)	−0.0293 (14)	−0.0432 (15)	0.0135 (12)
C4	0.091 (2)	0.098 (2)	0.095 (2)	−0.049 (2)	−0.062 (2)	0.0368 (19)
C5	0.0508 (13)	0.0620 (15)	0.0588 (14)	−0.0145 (11)	−0.0276 (11)	−0.0061 (11)
C6	0.0382 (10)	0.0364 (10)	0.0403 (10)	−0.0095 (8)	−0.0099 (8)	−0.0087 (8)
C7	0.0413 (10)	0.0343 (10)	0.0388 (10)	−0.0127 (8)	−0.0101 (8)	−0.0043 (8)
C8	0.0446 (11)	0.0389 (11)	0.0500 (12)	−0.0172 (9)	−0.0174 (9)	0.0005 (9)
C9	0.0739 (18)	0.0593 (15)	0.0632 (16)	−0.0208 (13)	−0.0333 (14)	0.0151 (12)
C10	0.0672 (19)	0.116 (3)	0.096 (2)	−0.0219 (19)	−0.0418 (18)	−0.017 (2)
C11	0.0476 (12)	0.0426 (11)	0.0474 (12)	−0.0156 (9)	−0.0152 (10)	−0.0088 (9)
C12	0.0610 (14)	0.0459 (12)	0.0575 (14)	−0.0215 (11)	−0.0209 (11)	−0.0061 (10)
C13	0.096 (2)	0.0687 (17)	0.0725 (18)	−0.0496 (17)	−0.0292 (16)	−0.0091 (14)
C14	0.0703 (17)	0.085 (2)	0.0742 (18)	−0.0492 (16)	−0.0134 (14)	−0.0111 (15)
C15	0.0485 (12)	0.0390 (11)	0.0502 (12)	−0.0131 (9)	−0.0140 (10)	−0.0070 (9)
C16	0.0689 (19)	0.0710 (19)	0.085 (2)	−0.0228 (15)	0.0167 (16)	−0.0416 (16)
C18	0.0558 (13)	0.0470 (12)	0.0408 (11)	−0.0238 (10)	−0.0114 (10)	0.0017 (9)
S3_1	0.0748 (7)	0.0432 (5)	0.0734 (8)	−0.0199 (5)	−0.0213 (6)	−0.0125 (4)
C19_1	0.0562 (12)	0.0403 (10)	0.0427 (10)	−0.0240 (9)	−0.0108 (9)	0.0021 (8)
C20_1	0.064 (2)	0.050 (2)	0.057 (2)	−0.0153 (16)	−0.0229 (19)	−0.0030 (19)
C21_1	0.070 (2)	0.055 (2)	0.0648 (18)	−0.0298 (17)	−0.0234 (18)	−0.0042 (15)
C22_1	0.066 (2)	0.061 (2)	0.0605 (19)	−0.0267 (16)	−0.0214 (17)	−0.0009 (18)
S3_2	0.059 (2)	0.059 (3)	0.069 (2)	−0.0261 (17)	−0.0281 (19)	0.002 (2)
C19_2	0.0562 (12)	0.0403 (10)	0.0427 (10)	−0.0240 (9)	−0.0108 (9)	0.0021 (8)
C20_2	0.057 (3)	0.048 (3)	0.051 (4)	−0.031 (3)	−0.012 (3)	0.002 (3)
C21_2	0.062 (4)	0.055 (4)	0.065 (4)	−0.030 (3)	−0.023 (3)	0.003 (3)
C22_2	0.061 (4)	0.051 (4)	0.059 (4)	−0.032 (3)	−0.019 (4)	0.002 (3)
C17_2	0.066 (4)	0.215 (11)	0.328 (15)	−0.037 (6)	0.012 (7)	−0.201 (11)
C17A_2	0.043 (7)	0.058 (7)	0.018 (4)	−0.021 (6)	0.012 (4)	−0.009 (5)

### Geometric parameters (Å, º)

**Table:**

S1—O2	1.4270 (17)	C11—C12	1.387 (3)
S1—O1	1.4324 (16)	C12—C13	1.422 (4)
S1—C7	1.808 (2)	C12—H12	0.9300
S1—C1	1.813 (2)	C13—C14	1.335 (4)
S2—C14	1.694 (3)	C13—H13	0.9300
S2—C11	1.715 (2)	C14—H14	0.9300
O3—C8	1.191 (3)	C16—C17_2	1.356 (10)
O4—C8	1.324 (3)	C16—C17A_2	1.489 (19)
O4—C9	1.453 (3)	C16—H16	0.9300
O5—C15	1.189 (3)	C16—H16A	0.9700
O6—C15	1.314 (3)	C16—H16B	0.9700
O6—C16	1.455 (3)	C18—C19_2	1.499 (3)
N1—C6	1.461 (3)	C18—C19_1	1.499 (3)
N1—C2	1.463 (3)	C18—H18A	0.9700
N1—C5	1.475 (3)	C18—H18B	0.9700
N1—H1	0.9800	C18—H18C	0.9700
C1—C15	1.527 (3)	C18—H18D	0.9700
C1—C18	1.546 (3)	S3_1—C19_1	1.705 (2)
C1—C2	1.551 (3)	S3_1—C21_1	1.728 (4)
C2—C3	1.531 (3)	C19_1—C20_1	1.341 (6)
C2—H2	0.9800	C20_1—C22_1	1.430 (7)
C3—C4	1.486 (4)	C20_1—H20A_1	0.9700
C3—H3A	0.9700	C20_1—H20B_1	0.9700
C3—H3B	0.9700	C21_1—C22_1	1.332 (5)
C4—C5	1.512 (4)	C21_1—H21_1	0.9300
C4—H4A	0.9700	C22_1—H22_1	0.9300
C4—H4B	0.9700	S3_2—C19_2	1.704 (2)
C5—H5A	0.9700	S3_2—C21_2	1.728 (4)
C5—H5B	0.9700	C19_2—C20_2	1.341 (6)
C6—C11	1.503 (3)	C20_2—C22_2	1.430 (7)
C6—C7	1.537 (3)	C20_2—H20_2	0.9300
C6—H6	0.9800	C21_2—C22_2	1.332 (5)
C7—C8	1.521 (3)	C21_2—H21_2	0.9300
C7—H7	0.9800	C22_2—H22_2	0.9300
C9—C10	1.463 (4)	C17_2—H17A_2	0.9600
C9—H9A	0.9700	C17_2—H17B_2	0.9600
C9—H9B	0.9700	C17_2—H17C_2	0.9600
C10—H10A	0.9600	C17A_2—H17D_2	0.9600
C10—H10B	0.9600	C17A_2—H17E_2	0.9600
C10—H10C	0.9600	C17A_2—H17F_2	0.9600
			
O2—S1—O1	119.13 (11)	C11—C12—C13	110.5 (2)
O2—S1—C7	109.00 (10)	C11—C12—H12	124.7
O1—S1—C7	106.71 (10)	C13—C12—H12	124.7
O2—S1—C1	110.41 (10)	C14—C13—C12	114.2 (2)
O1—S1—C1	106.16 (10)	C14—C13—H13	122.9
C7—S1—C1	104.40 (10)	C12—C13—H13	122.9
C14—S2—C11	92.13 (13)	C13—C14—S2	112.0 (2)
C8—O4—C9	117.8 (2)	C13—C14—H14	124.0
C15—O6—C16	117.5 (2)	S2—C14—H14	124.0
C6—N1—C2	113.82 (16)	O5—C15—O6	124.4 (2)
C6—N1—C5	112.22 (17)	O5—C15—C1	121.9 (2)
C2—N1—C5	104.09 (17)	O6—C15—C1	113.64 (18)
C6—N1—H1	108.8	C17_2—C16—O6	111.8 (4)
C2—N1—H1	108.8	O6—C16—C17A_2	109.3 (7)
C5—N1—H1	108.8	C17_2—C16—H16	124.1
C15—C1—C18	110.35 (17)	O6—C16—H16	124.1
C15—C1—C2	111.72 (18)	O6—C16—H16A	109.8
C18—C1—C2	108.07 (17)	C17A_2—C16—H16A	109.8
C15—C1—S1	112.48 (14)	O6—C16—H16B	109.8
C18—C1—S1	107.65 (15)	C17A_2—C16—H16B	109.8
C2—C1—S1	106.34 (14)	H16A—C16—H16B	108.3
N1—C2—C3	103.90 (19)	C19_2—C18—C1	118.81 (18)
N1—C2—C1	112.87 (16)	C19_1—C18—C1	118.81 (18)
C3—C2—C1	114.40 (19)	C19_1—C18—H18A	107.6
N1—C2—H2	108.5	C1—C18—H18A	107.6
C3—C2—H2	108.5	C19_1—C18—H18B	107.6
C1—C2—H2	108.5	C1—C18—H18B	107.6
C4—C3—C2	105.1 (2)	H18A—C18—H18B	107.0
C4—C3—H3A	110.7	C19_2—C18—H18C	107.6
C2—C3—H3A	110.7	C1—C18—H18C	107.6
C4—C3—H3B	110.7	C19_2—C18—H18D	107.6
C2—C3—H3B	110.7	C1—C18—H18D	107.6
H3A—C3—H3B	108.8	H18C—C18—H18D	107.0
C3—C4—C5	106.6 (2)	C19_1—S3_1—C21_1	91.52 (18)
C3—C4—H4A	110.4	C20_1—C19_1—C18	127.1 (3)
C5—C4—H4A	110.4	C20_1—C19_1—S3_1	109.6 (3)
C3—C4—H4B	110.4	C18—C19_1—S3_1	122.94 (17)
C5—C4—H4B	110.4	C19_1—C20_1—C22_1	116.2 (5)
H4A—C4—H4B	108.6	C19_1—C20_1—H20A_1	108.2
N1—C5—C4	103.6 (2)	C22_1—C20_1—H20A_1	108.2
N1—C5—H5A	111.0	C19_1—C20_1—H20B_1	108.2
C4—C5—H5A	111.0	C22_1—C20_1—H20B_1	108.2
N1—C5—H5B	111.0	H20A_1—C20_1—H20B_1	107.4
C4—C5—H5B	111.0	C22_1—C21_1—S3_1	113.4 (4)
H5A—C5—H5B	109.0	C22_1—C21_1—H21_1	123.3
N1—C6—C11	110.30 (17)	S3_1—C21_1—H21_1	123.3
N1—C6—C7	110.96 (16)	C21_1—C22_1—C20_1	109.0 (5)
C11—C6—C7	108.32 (17)	C21_1—C22_1—H22_1	125.5
N1—C6—H6	109.1	C20_1—C22_1—H22_1	125.5
C11—C6—H6	109.1	C19_2—S3_2—C21_2	85.3 (6)
C7—C6—H6	109.1	C20_2—C19_2—C18	123.6 (7)
C8—C7—C6	111.47 (16)	C20_2—C19_2—S3_2	114.4 (6)
C8—C7—S1	105.56 (14)	C18—C19_2—S3_2	121.4 (3)
C6—C7—S1	113.68 (14)	C19_2—C20_2—C22_2	114.6 (11)
C8—C7—H7	108.7	C19_2—C20_2—H20_2	122.7
C6—C7—H7	108.7	C22_2—C20_2—H20_2	122.7
S1—C7—H7	108.7	C22_2—C21_2—S3_2	120.2 (12)
O3—C8—O4	125.3 (2)	C22_2—C21_2—H21_2	119.9
O3—C8—C7	124.6 (2)	S3_2—C21_2—H21_2	119.9
O4—C8—C7	110.05 (18)	C21_2—C22_2—C20_2	103.9 (13)
O4—C9—C10	111.5 (2)	C21_2—C22_2—H22_2	128.1
O4—C9—H9A	109.3	C20_2—C22_2—H22_2	128.1
C10—C9—H9A	109.3	C16—C17_2—H17A_2	109.5
O4—C9—H9B	109.3	C16—C17_2—H17B_2	109.5
C10—C9—H9B	109.3	H17A_2—C17_2—H17B_2	109.5
H9A—C9—H9B	108.0	C16—C17_2—H17C_2	109.5
C9—C10—H10A	109.5	H17A_2—C17_2—H17C_2	109.5
C9—C10—H10B	109.5	H17B_2—C17_2—H17C_2	109.5
H10A—C10—H10B	109.5	C16—C17A_2—H17D_2	109.5
C9—C10—H10C	109.5	C16—C17A_2—H17E_2	109.5
H10A—C10—H10C	109.5	H17D_2—C17A_2—H17E_2	109.5
H10B—C10—H10C	109.5	C16—C17A_2—H17F_2	109.5
C12—C11—C6	128.0 (2)	H17D_2—C17A_2—H17F_2	109.5
C12—C11—S2	111.20 (18)	H17E_2—C17A_2—H17F_2	109.5
C6—C11—S2	120.78 (15)		
			
O2—S1—C1—C15	40.84 (18)	N1—C6—C11—C12	−124.6 (2)
O1—S1—C1—C15	171.28 (15)	C7—C6—C11—C12	113.8 (3)
C7—S1—C1—C15	−76.17 (17)	N1—C6—C11—S2	55.8 (2)
O2—S1—C1—C18	−80.94 (16)	C7—C6—C11—S2	−65.8 (2)
O1—S1—C1—C18	49.50 (17)	C14—S2—C11—C12	−0.3 (2)
C7—S1—C1—C18	162.05 (14)	C14—S2—C11—C6	179.4 (2)
O2—S1—C1—C2	163.44 (14)	C6—C11—C12—C13	−179.5 (2)
O1—S1—C1—C2	−66.12 (16)	S2—C11—C12—C13	0.2 (3)
C7—S1—C1—C2	46.43 (16)	C11—C12—C13—C14	−0.1 (4)
C6—N1—C2—C3	−162.13 (19)	C12—C13—C14—S2	−0.1 (4)
C5—N1—C2—C3	−39.6 (2)	C11—S2—C14—C13	0.2 (3)
C6—N1—C2—C1	73.4 (2)	C16—O6—C15—O5	0.4 (4)
C5—N1—C2—C1	−164.14 (18)	C16—O6—C15—C1	178.5 (2)
C15—C1—C2—N1	60.9 (2)	C18—C1—C15—O5	−27.1 (3)
C18—C1—C2—N1	−177.54 (17)	C2—C1—C15—O5	93.1 (3)
S1—C1—C2—N1	−62.20 (19)	S1—C1—C15—O5	−147.4 (2)
C15—C1—C2—C3	−57.7 (3)	C18—C1—C15—O6	154.7 (2)
C18—C1—C2—C3	63.9 (2)	C2—C1—C15—O6	−85.0 (2)
S1—C1—C2—C3	179.27 (18)	S1—C1—C15—O6	34.5 (3)
N1—C2—C3—C4	24.3 (3)	C15—O6—C16—C17_2	−118.1 (7)
C1—C2—C3—C4	147.8 (2)	C15—O6—C16—C17A_2	−141.0 (7)
C2—C3—C4—C5	−0.2 (4)	C15—C1—C18—C19_2	−66.3 (2)
C6—N1—C5—C4	163.0 (2)	C2—C1—C18—C19_2	171.31 (19)
C2—N1—C5—C4	39.5 (3)	S1—C1—C18—C19_2	56.8 (2)
C3—C4—C5—N1	−23.8 (3)	C15—C1—C18—C19_1	−66.3 (2)
C2—N1—C6—C11	175.34 (17)	C2—C1—C18—C19_1	171.31 (19)
C5—N1—C6—C11	57.4 (2)	S1—C1—C18—C19_1	56.8 (2)
C2—N1—C6—C7	−64.6 (2)	C1—C18—C19_1—C20_1	−107.6 (4)
C5—N1—C6—C7	177.47 (18)	C1—C18—C19_1—S3_1	80.6 (2)
N1—C6—C7—C8	171.04 (16)	C21_1—S3_1—C19_1—C20_1	2.3 (4)
C11—C6—C7—C8	−67.7 (2)	C21_1—S3_1—C19_1—C18	175.3 (3)
N1—C6—C7—S1	51.9 (2)	C18—C19_1—C20_1—C22_1	−178.0 (4)
C11—C6—C7—S1	173.09 (13)	S3_1—C19_1—C20_1—C22_1	−5.4 (7)
O2—S1—C7—C8	75.37 (16)	C19_1—S3_1—C21_1—C22_1	1.4 (4)
O1—S1—C7—C8	−54.49 (16)	S3_1—C21_1—C22_1—C20_1	−4.4 (7)
C1—S1—C7—C8	−166.65 (14)	C19_1—C20_1—C22_1—C21_1	6.4 (8)
O2—S1—C7—C6	−162.15 (14)	C1—C18—C19_2—C20_2	89.6 (9)
O1—S1—C7—C6	67.99 (16)	C1—C18—C19_2—S3_2	−99.5 (3)
C1—S1—C7—C6	−44.17 (16)	C21_2—S3_2—C19_2—C20_2	−0.9 (13)
C9—O4—C8—O3	−6.6 (3)	C21_2—S3_2—C19_2—C18	−172.5 (8)
C9—O4—C8—C7	172.04 (19)	C18—C19_2—C20_2—C22_2	−179.9 (11)
C6—C7—C8—O3	−22.8 (3)	S3_2—C19_2—C20_2—C22_2	8.6 (17)
S1—C7—C8—O3	101.0 (2)	C19_2—S3_2—C21_2—C22_2	−8.0 (19)
C6—C7—C8—O4	158.49 (17)	S3_2—C21_2—C22_2—C20_2	13 (2)
S1—C7—C8—O4	−77.62 (19)	C19_2—C20_2—C22_2—C21_2	−13 (2)
C8—O4—C9—C10	−75.7 (3)		

### Hydrogen-bond geometry (Å, º)

**Table:**

*D*—H···*A*	*D*—H	H···*A*	*D*···*A*	*D*—H···*A*
C10—H10*B*···O2^i^	0.96	2.63	3.227 (4)	120
C2—H2···O1^ii^	0.98	2.67	3.519 (3)	145
C5—H5*A*···O1^ii^	0.97	2.69	3.491 (3)	141
C6—H6···O1^ii^	0.98	2.70	3.560 (3)	146

Symmetry codes: (i) −*x*, −*y*+2, −*z*; (ii) −*x*, −*y*+2, −*z*+1.
